# Ecological Preferences and Diversity of Essential Oil Composition in Endangered Wild-Growing Populations of *Sideritis sipylea* Boiss. (Lamiaceae) of the East Aegean Islands (Greece): Evidencing Antioxidant Potential, Antimicrobial and Cytotoxic Activities

**DOI:** 10.3390/plants12040836

**Published:** 2023-02-13

**Authors:** Pavle Z. Mašković, Rafaela Stagiopoulou, Nemanja Miletić, Nikos Krigas, Diamanto Lazari

**Affiliations:** 1 Department of Chemistry and Chemical Engineering Faculty of Agronomy, University of Kragujevac, Cara Dušana 34, 32000 Čačak, Serbia; 2 Laboratory of Pharmacognosy, School of Pharmacy, Faculty of Health Sciences, Aristotle University of Thessaloniki, 54124 Thessaloniki, Greece; 3 Institute of Plant Breeding and Genetic Resources, Hellenic Agricultural Organization Demeter, Thermi, 57001 Thessaloniki, Greece

**Keywords:** GC-MS analysis, DPPH, ABTS, bacteria, fungi, cell lines, biological activity, ecological profile, threatened plants

## Abstract

Plants from the genus *Sideritis* (Lamiaceae) have been widely used in folk medicine for a long time and consequently are a focus of the scientific community. Despite this interest, explicit data about the essential oils (EOs) of the Endangered *Sideritis sipylea* have not been readily available to date. In this study, we investigated the ecological preferences of Greek *S. sipylea* and the chemical composition of the essential oils of wild-growing *S. sipylea* populations from two Greek islands (S1: Samos, S2: Lesvos); we explored concomitant associations with environmental factors; and we assessed their (i) antioxidant potential (two tests), (ii) antimicrobial activity against six microbial and two fungal strains, and (iii) cytotoxic effect in two human and one murine cell lines. We compiled an ecological profile in R based on all known Greek localities of *S. sipylea*, outlining for the first time its preferences regarding temperature (3.48 ± 1.53 °C to 30.70 ± 1.11 °C) and the precipitation regimes (5.92 ± 2.33 mm to 136 ± 11.43 mm) shaping its natural occurrence in the wild. The chemical analysis (42 compounds in total) confirmed the domination of monoterpene hydrocarbons in both samples (with quantitative and qualitative differences) and identified 12 new constituents reported in *S. sipylea* for the first time (e.g., Bicyclogermacrene and Cumacrene). Dominant compounds in S1 (39 constituents) were *β*-Myrcene (20.4%) followed by *β*-caryophyllene (11.8%), bicyclogermacrene (7.1%), *β*-pinene (6.3%), carvacrol (6.2%) and *α*-terpinene (6.1%), whereas in S2 (26 constituents) the main ones were *α*-pinene (37.3%), *β*-pinene (15.1%) and sabinene (12.1%), followed by *β*-caryophyllene (5.6%) and bicyclogermacrene (5.5%). The strong antioxidant capacity and cytotoxic activity of *S. sipylea* EOs are reported herein for the first time, while new insight is provided regarding their effect on bacterial and fungal strains (four ones originally tested herein). The biological activity analysis demonstrated variation among samples, with S2 being more potent than S1. Altogether, the results of the present study demonstrate the high biological potential of *S. sipylea* EOs with an interesting antioxidant capacity and antimicrobial and cytotoxic effects and reveal associations of natural chemodiversity with climatic factors.

## 1. Introduction

Essential oils (EOs) represent a mixture of volatile compounds, mainly of mono- and sesquiterpenoids, benzenoids and phenylpropanoids among others and represent some of the many plant products targeted by the scientific community due to strong biological properties allowing important industrial applications [1]. The EOs chemical compositions, as well as their antioxidant and antimicrobial properties, affect various physiological processes in both plant and human organisms, exerting beneficial properties, such as free radical scavenging activity and pathogen growth inhibition [2]. Deep interest has been developed in the research of EOs [3,4,5] due to the resistance of some bacteria and fungi to the currently commercially available synthetic antibiotics or due to the occurrence of side effects during antibiotics application. Among the wide range of biological activities of the EOs, recent studies have demonstrated specific antimicrobial, antiviral, antioxidant, anticancer and hepatoprotective effects [6,7,8]. The ability of an aromatic medicinal plant bearing EOs to adapt to its habitat is closely related to responses to environmental challenges and stresses, either non-biotic (e.g., climate and soil properties) or biotic (e.g., herbivores, pathogens, and other microorganisms or pollination vectors) (e.g., [9]).

Natural antioxidant compounds with small molecular weights are considered possible solutions in the case of a failure of internal human enzymatic mechanisms and/or due to their inadequate efficiency [10]. Naturally occurring antioxidant compounds have been suggested as suitable replacements for synthetic ones, such as butylhydroxyanisole (BHA) and butylhydroxytoluene (BHT) [11]. There is also a growing interest in the naturally occurring antioxidant compounds that may be directly consumed through food consumption (e.g., herbs, vegetables and fruits) or as pharmaceuticals as well [12]. Natural phenolic compounds have been shown to provide a prolific defense against the oxidative stress from free radicals and oxidizing agents, even in natural conditions [12,13,14]. It has been proved that most of the herbal infusions that are traditionally used in folk medicine usually exhibit significant antioxidant and other pharmacological properties, which are usually attributed to the presence of phenolic compounds, especially flavonoids. This class of compounds is also known for its ability to prevent the oxidation of fatty acids, thus providing an additional value to the plants, which have been used as food or fodder [15].

Different species and subspecies of the genus *Sideritis* have been widely used as traditional herbal teas in Greece (Greek mountain tea), the Balkans (shepherd’s tea), and Turkey (Turkish mountain tea) as well as in other Mediterranean countries [16]. Therefore, several plants of this genus are widely used as nervous system stimulants or as anti-inflammatory, antispasmodic, carminative, analgesic, sedative, antitussive, stomachic and anticonvulsant agents, in the treatment of coughs due to colds and for curing gastrointestinal disorders [16,17,18], while their constituents exhibit strong antimicrobial, antiseptic, anti-inflammatory, antirheumatic activities and insecticidal properties [19,20]. The scientific name of the genus *Sideritis* actually originates from the Greek word “Sideron”, due to its alleged ability to promote the healing of wounds caused by iron blades in ancient times [21]. This genus belongs to the Lamiaceae (Labiatae) family, with over 150 species found in the Mediterranean area [22]. 

Despite the great interest [16] in the study of several mountain tea plants (*Sideritis* spp.), the current knowledge about the essential oils of *Sideritis sipylea* Boiss. and their biological activities is still rather limited [17,23,24,25,26,27,28,29,30,31]. Some studies have focused on the phytochemical profile [32], biological activity [32], polar constituents [33], and nutritional value [33] of *S. sipylea*. Although the ecological preferences of other Greek endemic *Sideritis* spp. have been evaluated recently [34], detailed information about the temperature or precipitation preferences of the wild-growing populations of *S. sipylea* have not been investigated yet. In the same fashion, the EOs composition of wild-growing plants with regard to environmental factors have been investigated before [9,35]; however, no such data exist regarding the chemical composition of *S. sipylea* EOs associated with environmental factors. In fact, *S. sipylea* is an endemic species of some islands of the East Aegean Archipelago such as Lesvos (Mt. Olympus), Samos (Mt. Kerkis or Ambelos), Chios (Mt. Pelineon and Mt. Kochlias) and Ikaria (Mt. Atheras); additionally, it has also been found in small parts of the adjacent Anatolia region in Turkey [36]. In the Greek East Aegean islands, it is naturally found from 250 to 1.200 m above sea level while its Anatolian populations may occur up to 1.600 m. It is assessed as an Endangered species in Greece due to overgrazing and uncontrolled overcollection, geographical isolation and restricted gene flow among its populations located in four islands along the East Aegean Archipelago [36]. 

In this study, we analyzed the EOs of the endangered *S. sipylea* based on material sourced from two wild-growing populations in different Greek islands (Samos, Lesvos), thus using material from the most northern and most southern distribution limits in Greece. We assessed its EOs in terms of antioxidant potential with two different tests, we investigated their antimicrobial activity against six microbial and two fungal strains, and we examined their cytototoxic activity in one murine and two human cell lines. Attempting to determine the local climatic conditions for this endangered species, we further investigated the ecological preferences of all currently known Greek populations of *S. sipylea.* To this end, we compiled all respective data about monthly temperature, monthly precipitation and another 19 bioclimatic variables in an ecological profile using R, and we further compared the habitat conditions of the two sampled populations with an independent sample *t*-test.

## 2. Results and Discussion

### 2.1. Chemical Profile of the Essential Oil of S. sipylea

**Figure 1:**
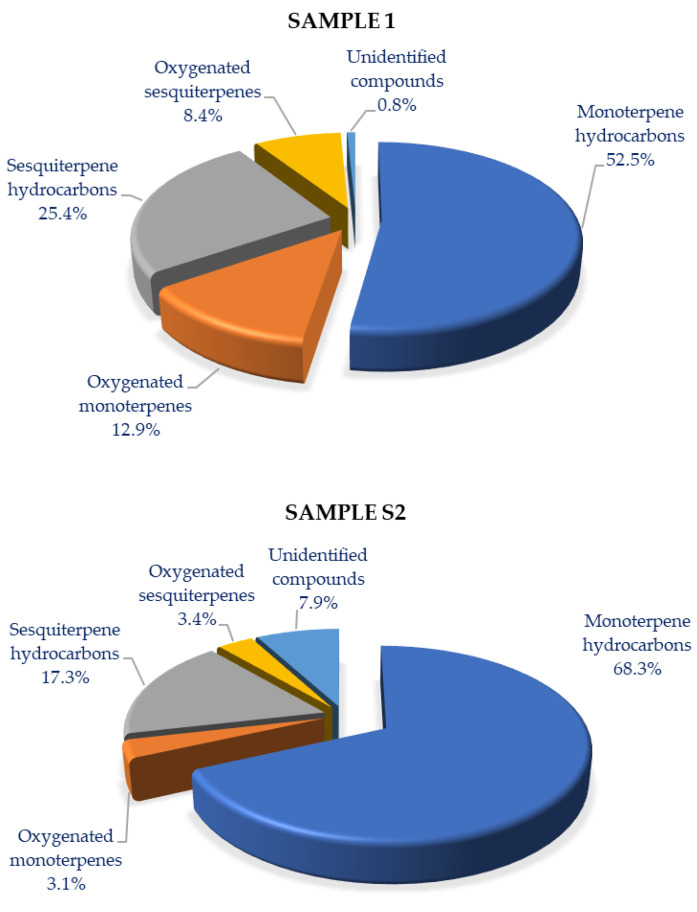
Fractions of terpenoid classes in the samples of wild-growing *Sideritis sipylea* populations from Greece (S1: mid-summer collection, Samos Island; S2: early summer collection, Lesvos Island).

The yields of the volatile fractions obtained from *S. sipylea* from Mt Kerkis, Samos Island (S1: collected at the peak of flowering) and Mt Olympus in Lesvos Island (S2: collected at the beginning of flowering) were 0.43% and 0.44%, respectively, on a dry weight basis. The chemical profiles of the isolated EOs of the studied *S. sipylea* samples from Greece are presented in Table 1, while fractions of the detected terpenoid classes are given in Figure 1. 

To date, the knowledge on the EO composition of *S. sipylea* is restricted in a single report from wild-growing populations of Samos Island, Greece [23], which is supplemented by general information in a review of the EOs of 50 Turkish *Sideritis* spp. [17]. Our study provides a detailed report on the chemical composition of the EOs of *S. sipylea* from two Greek Islands (Samos and Lesvos; material from the latter island is studied for the first time). Although only two samples of *S. sipylea* were examined herein, it should be mentioned that these represent 40% of its global distribution and half of its natural range in Greece (occurrence in four islands), originating from its most northern and most southern distribution limits in Greece [36]. In total, 42 different compounds were detected in the EOs extracted from the two Greek populations of *S. sipylea* (Table 1), among which 12 new constituents such as Bicyclogermacrene and Cumacrene are reported herein for the first time [23]. In comparison to previous studies [23], the studied Greek samples share 29 common constituents (Table 1), whereas 50 constituents previously reported in other studies [17,23] have not been identified in our study (e.g., *β*-elemene, cryptone; the rest were compounds with a proportion <0.5%). Considering that the S1 sample was collected at the peak of flowering as in previous studies [23] and the S2 sample was sourced at the beginning of flowering, this means that *S. sipylea* EO may be expected to include in total more than 90 different constituents throughout its flowering period.

Among the compounds identified in the studied Greek samples, 23 were present in both populations (Table 1), 16 were detected only in the mid-July sample S1 originating from Mt Kerkis (Samos Island), and 3 compounds were detected only in the early summer sample originating from Mt Olympus, Lesvos Island (S2 sample). Thus, the essential oil of the mid-summer S1 sample (39 constituents) was found to be more diverse in comparison to the early summer S2 sample (26 constituents).

*S. sipylea* EOs are known to be monoterpene-hydrocarbon-rich and especially myrcene-rich and pinene-rich oils [17]. The dominant constituents in the mid-summer S1 sample (Mt Kerkis, Samos Island) were *β*-myrcene (20.4%), followed by *β*-caryophyllene (11.8%), bicyclogermacrene (7.1%), *β*-pinene (6.3%), carvacrol (6.2%) and *α*-terpinene (6.1%). In the case of the early summer S2 sample (Mt Olympus, Lesvos Island), the main compounds were *α*-pinene (37.3%), *β*-pinene (15.1%) and sabinene (12.1%), followed by *β*-caryophyllene (5.6%) and bicyclogermacrene (5.5%). The two studied samples originating in different Greek islands presented different main constituents in their EOs. For example, the dominant role of *β*-myrcene and the noticeable proportion of carvacrol in the mid-summer S1 sample (20.4% and 6.2%, respectively) were both reduced remarkably in the early summer S2 sample (0.6% and 0.1%, respectively); *β*-caryophyllene was more pronounced in mid-summer S1 (11.7%) in comparison to the early summer S2 sample (5.6%); *α*-pinene, *β*-pinene and sabinene were more pronounced in the early summer S2 sample (37.3%, 15.1% and 12.1%%, respectively) than in the mid-summer S1 sample (4.3%, 6.3% and 0.5%, respectively). These qualitative and quantitative differences between the individual volatiles of the distilled oils of the studied *S. sipylea* populations may be expected as the composition of the EOs from many *Sideritis* spp. has been reported to be largely influenced by geographical location, season and collection date [16,37].

It might be noticed that, in both *S. sipylea* Greek samples, monoterpene hydrocarbons were the dominant class in the studied EOs (Figure 1) and their content was 68.3% in S2 and 52.5% in S1. The fraction of the rest of the terpenoid classes was higher in the mid-summer S1 sample, while this difference was mostly notable in the case of oxygenated monoterpenes (12.9 versus 3.1% for S1 and S2, respectively). It might be also referred that the content of unidentified compounds was higher in the early summer S2 sample than in the mid-summer S1 sample (7.9% versus 0.8%, respectively).

Our results confirmed previous claims that monoterpene hydrocarbons usually represent the main constituents of the EOs of several members of the genus *Sideritis* originating from Turkey, Greece or Spain [16,17,38,39,40,41]. As these constituents are also recognized for their characteristic odor and taste, they find their application in the food and cosmetic industries and are also used as insecticides, insect repellents or drug attractants [1]. These compounds express a wide range of biological activities (anticancer, antinociceptive, antiviral, antiphlogistic and antioxidant) as well as beneficial effect on risk factors for cardiovascular disease [42,43].

### 2.2. Biological Activities of the Essential Oil of S. sipylea

The biological activity of the isolated EOs of *S. sipylea* was assessed using antioxidant, cytotoxic and antimicrobial assays. The antioxidant activity was determined against DPPH and ABTS radical scavenging (Table 2).

Although the antioxidant activity of several *Sideritis* spp. is reported in the literature [44], there is no study assessing the antioxidant capacity of *S. sipylea* EOs. In our study, the results showed that the early summer S2 sample was more potent in the case of both DPPH and ABTS radicals scavenging. Indeed, sample S2 expressed higher activity against the DPPH radical compared to the synthetic antioxidant BHT. However, the same sample (S2) exerted slightly higher activity against the ABTS radical in comparison to the ascorbic acid (AA). In summary, both tested Greek *S. sipylea* samples exhibited high levels of antioxidant activity, whereas only a small amount of the sample was enough to neutralize radical scavenging. Previous studies assessing the antioxidant activity of lyophilized aqueous, methanol, ethanol or acetone extracts of Turkish *S. sipylea* [25,26] have also demonstrated a strong antioxidant profile (IC_50_: 0.05 to 1.1 mg/mL), thus rendering it an effective natural antioxidant [26].

The antimicrobial activity of the EOs of Greek *S. sipylea* samples was tested against six microbial and two fungal strains and the obtained results are presented in Table 3. 

The highest activity against the selected bacteria was exhibited by the mid-summer S2 sample against *Staphylococcus aureus* (MIC: 6.25 µg/mL). The sample S1 expressed the strongest activity against the fungus *Candida albicans* (MIC: 3.12 µg/mL), while the activity of the S2 sample against the same strain was slightly higher. Summarizing the activity of both samples against all tested strains, it might be concluded that the mid-summer sample S2 proved to be slightly more potent than S1, which is in line with the results of antioxidant activity assays.

Our study reports for the first time the effects of the EO of S. sipylea on specific bacteria such as Proteus hauseri, P. mirabilis and Klabsiella pneumoniae and on the fungus Aspergillus niger. In previous studies aiming to determine the constituents of the essential oil of S. sipylea that are responsible for its intense antimicrobial activity, the different fractions varied in their antimicrobial activity against Escherischia coli (ATCC 10536), P. aeruginosa (ATCC 9027), S. aureus (ATCC 6538), Bacillus cereus (ATCC 11778), B. subtilis (ATTCC 6633), Micrococcus luteus (ATCC 9341) and C. albicans (ATCC 10231), while the fractions with the highest activity were those with alcohols as main constituents [24]. The methanol extracts from the aerial parts of wild-growing Turkish S. sipylea were found to exhibit antifungal activity against the clotrimazole-resistant C. albicans, thus suggesting a potential to be used for candidiasis treatment. However, this effect was weaker in comparison to other Turkish Sideritis spp. such as S. trojana Bornm. [45]. Some isolated diterpenes (including siderol, linearol and epicandicandiol) and diacetate derivatives of linearol and epicandicandiol from wild Turkish S. sipylea plants have not been found active against bacteria such as S. aureus (ATTC 25923), B. subtilis (ATCC 6633), E. coli (ATCC 11230) and Pseudomonas aeruginosa (ATCC 27853) and the fungus C. albicans (ATCC 90028), thus showing little potential as effective drugs [31]. The only active compound reported to date against S. aureus, B. subtilis and C. albicans has been epicandicandiol, along with the presence of an acetyl group occurring in siderol and linearol, which appears to be related to decreased activity.

Previously conducted studies have shown that the chemical structures of the presented compounds of EOs play an important role in their biological activities. It has been proved that the hydroxyl groups express a significant influence on the antioxidant and antimicrobial activities, whereas the compounds with the hydroxyl groups may express more potent activity than the compounds with the carbonyl groups, under the negligible influence of double bonds and acyclic, bicyclic and/or monocyclic structures [46,47,48,49,50]. The lipophilicity and hydrophobicity of the monoterpenes, which constitute the consequence of their chemical structures, allow them to penetrate into the cell membrane and to interact with the phospholipids causing membrane expansion, increasing fluidity and permeability, the disturbance of membrane proteins, the inhibition of the respiratory process and alterations in ion transport [2,3,4,5,6,7]. Such alterations may induce further changes leading into the leakage of intracellular materials [46]. In such circumstances, it might be concluded that both the hydroxyl group and the lipophilicity of the monoterpenes are responsible for their antimicrobial activity.

The cytotoxic activity of the essential oil samples of *S. sipylea* was assessed using three different cell lines, i.e., Hep2c, RD and L2OB (Table 4). 

Despite the great interest regarding the biological activities of Sideritis spp., there are only scarce data regarding their cytotoxic effects [51]. It is important to mention that our study provides the first report on the specific cytotoxic effects of the essential oils of S. sipylea. The results herein showed that the S1 sample expressed higher cytotoxic activity in the case of Hep2c and L2OB cell lines, while the opposite was evidenced in the case of the RD cell line. In terms of the basic criterion for the cytotoxic activity of a plant extract (i.e., activity <30 µg/mL), the results herein demonstrated that this was fulfilled in the case of the activity of the mid-summer S1 sample against Hep2c and L2OB cell lines as well as in the case of the early summer S2 sample against RD and L2OB cell lines. The possible reason for such differences in the sensitivity of the tested cell lines in the case of S. sipylea EOs may be the diversity in the chemical composition of the samples examined, possibly in the same fashion with their antioxidant and antimicrobial activities.

### 2.3. Ecological Profiling of Sideritis sipylea

**Figure 2:**
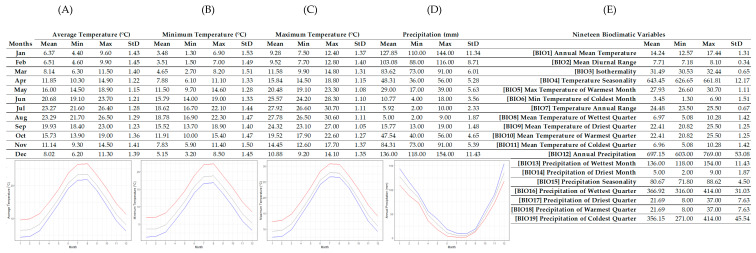
R-derived ecological profile of the wild-growing populations of *Sideritis sipylea* based on thirteen known Greek localities (N = 13) with WorldClim (version 2.1) data-mined values (minimum, maximum, average and standard deviation) for temperature (**A**–**C**), precipitation (**D**) and 19 bioclimatic variables (**E**). Line graphs (**A**–**C**) illustrate the minimum (blue), maximum (red) and mean (gray) monthly temperature (°C). Line graph (**D**) illustrates the minimum (red), maximum (blue) and mean (gray) monthly precipitation (mm).

The ecological preferences of *Sideritis sipylea* in terms of climate conditions were compiled in Figure 2. The ecological profile was based on 80% of its global distribution records and all its known Greek localities [36] showing that *S. sipylea*’s wild-growing populations may only appear in sites with an average annual temperature of 14.24 ± 1.31 °C and in areas with an annual precipitation of 697.15 ± 53.08 mm. The highest observed temperatures in those areas were in July (27.92 ± 1.11 °C) and in August (27.78 ± 1.11 °C), with the highest value being 30.70 °C (natural upper temperature limit). The lowest observed temperatures were in January (3.48 ± 1.53 °C) and February (3.51 ± 1.49 °C), with the lowest value reaching 1.30 °C (natural lower temperature limit). The *S. sipylea*’s seasonal temperature pattern is probably related to the average temperatures in its distribution areas, which slowly start to increase from February (6.37 ± 1.43 °C), peaking in August (23.29 ± 1.29 °C); then, average temperatures start decreasing progressively to reach the lowest values in January (6.37 ± 1.43 °C). The highest precipitation mean in those areas was observed in December (136 ± 11.43 mm) and the highest value that could be reached potentially was 154 mm (natural upper precipitation limit), while the lowest precipitation mean was observed in August (5 ± 1.87 mm) with the lowest value of 2 mm (natural lower precipitation limit). This profiling may outline the natural adaptions of *S. sipylea* populations in terms of extreme limits in terms of temperature and precipitation (Table 5). Compared to *S. euboea* (another *Sideritis* species with a documented ecological profile) [34], *S. sipylea* appears to prefer higher summer temperatures and lower winter temperatures, enjoying higher precipitation throughout the year. It is likely assumed that these environmental conditions further reflect variations in the chemical composition of its EOs. 

### 2.4. Habitat Comparison for the Two Samples

The independent sample *t*-test showed that several environmental factors are statistically different with *p* < 0.05 between the two studied *S. sipylea* populations from the Samos and Lesvos islands. The observed differences were mainly related to precipitation of the wettest month, precipitation seasonality and the precipitation of the wettest and coldest quarters, as well as the precipitation regimes in different months (January, February, March, July, October and December). The rest of the bioclimatic variables had *p* > 0.05, thus outlining that the rest of the bioclimatic variables had no significant statistical differences in the cases of the Lesvos and Samos islands. These results are further confirmed by the individual values of the environmental factors used in the *t*-test, which show noteworthy and clearly visible arithmetic differences between the islands of Samos and Lesvos. The results from the independent sample *t*-test for the statistically significant results are shown in Supplementary Material Table S1. Additionally, in the Supplementary Material Table S2, we have included the mean values for each one of the environmental variables regarding the Samos and Lesvos islands, which validate the results of the independent samples *t*-test that has been performed.

Although the small sample size herein (two samples) cannot support robust statistics, several differences can be highlighted in terms of major compounds (defined as >1% content in at least one sample) as well as for minor compounds (concentrations <1%) in the studied essential oils of *S. sipylea* with regard to the respective local environmental data prevailing in the two Greek islands. We observed that the higher the values in some environmental factors (mean diurnal range, isothermality, temperature seasonality, temperature annual range, precipitation of the driest month and driest quarter, precipitation of the warmest quarter, precipitation from April to August), the higher the chemical compound concentration in both examined samples for some major ones (*α*-Pinene, Sabinene, *β*-Pinene, Terpinen-4-ol) and some minor ones (*α*-Campholenal, *cis*-3-Hexenyl isovalerate, Pulegone, *trans-β*-Farnesene and *β*-Bisabolene). For all these compounds, the lower the values in some other environmental factors (annual mean temperature, highest temperature of the warmest month, lowest temperature of the coldest month, mean temperature of the wettest and driest quarters, mean temperature of the warmest and coldest quarters, annual precipitation, precipitation of the driest and wettest month, precipitation seasonality, precipitation of the coldest quarter, precipitation from October to March, highest and lowest temperatures in every month), the lower the chemical compound concentration for some major ones (all the above-mentioned and *α*-Bisabololly appearing in Sample 2 from Lesvos Island) and minor ones (all the above-mentioned and trans-Verbenol only appearing in Sample 2 from Lesvos Island). 

In the same line, the higher the values in some other environmental factors (annual mean temperature, highest temperature of the warmest month, lowest temperature of the coldest month, mean temperature of the driest and wettest quarters, mean temperature of the coldest and warmest quarters, annual precipitation, precipitation of the driest and wettest month, precipitation seasonality, precipitation of the coldest quarter, precipitation from October to March, lowest temperature in every month), the higher the chemical compound concentration was evidenced for major compounds in both examined samples, such as *β*-Myrcene, *α*-Terpinene, Limonene, *γ*-Terpinene, Carvacrol, *β*-Caryophyllene, Cumacrene, Bicyclogermacrene, Spathulenol and Caryophyllene oxide (also *α*-Phellandrene, *p*-Cymene, *β*-Phellandrene, Thymol and Ledol only appearing in the sample from Lesvos Island), as well as for minor compounds of both studied samples, such as *α*-Thujene, Terpinolene, *α*-Terpineol and Germacrene D (also Linalool, Santolinyl acetate, Carvone, Bornyl acetate, *α*-Ylangene, *β*-Bourbonene, *α*-Caryophyllene, *allo*-Aromadendrene, Viridiflorene, *δ*-Cadinene and *α*-Cadinol only appearing in the sample from Lesvos Island). For all these compounds, it was also showcased that the lower the values in some other environmental factors (mean diurnal range, isothermality, temperature seasonality, temperature annual range, precipitation of the driest month and driest quarter, precipitation of the warmest quarter, precipitation from April to August), the lower the chemical compound concentration was.

Perhaps the major compounds *α*-Pinene, Sabinene, *β*-Pinene and Terpinen-4-ol may be associated with wild-growing *S. sipylea* responses to water stress. Similarly, the major compounds *β*-Myrcene, *α*-Terpinene, Limonene, *γ*-Terpinene, Carvacrol, *β*-Caryophyllene, Cumacrene, Bicyclogermacrene, Spathulenol and Caryophyllene oxide may likely be related to the high temperature and drought stress experienced by *S. sipylea* wild-growing populations. The last assumption is also connected to the fact that, although the two studied EO samples were sourced from wild-growing material thriving in areas with similar temperature ranges, the sample from Samos Island (most southern distribution limit of *S. sipylea*) was linked with higher temperature values and it was also the most diverse sample in terms of different chemical compounds. For instance, Linalool, Santolinyl acetate, Carvone, Bornyl acetate, *α*-Ylangene, *β*-Bourbonene, *α*-Caryophyllene, *allo*-Aromadendrene, Viridiflorene, *δ*-Cadinene and *α*-Cadinol were all compounds that appeared only in Sample 1 (even in very low concentration percentages), but they were not detected in Sample 2. 

In previous studies, assessing the effects of environmental stresses on EOs in other medicinal aromatic plants [52], *a*-Pinene was found to have a positive correlation to Zinc concentrations and Sabinene is assumed to be related to copper concentrations in *Mentha pulegium* L. [53]. Previous studies have shown that *β*-Myrcene in the EO of *Ocimum basilicum* L. as well as *γ*-Terpinene and Carvacrol in the EO of *Thymus carmanicus* L. were both found to be related to drought stress responses [54], which seems in agreement with our results and assumptions regarding the Greek *S. sipylea* EOs. However, there are no previous data about *S. sipylea* responses to temperature, drought or water stress to confirm such findings and observations. Further studies are certainly needed to possibly associate other major compounds detected in Greek *S. sipylea* (such as *β*-Pinene and Terpinen-4-ol, *α*-Terpinene, Limonene, *β*-Caryophyllene, Cumacrene, Bicyclogermacrene, Spathulenol and Caryophyllene oxide) with temperature, drought or water stress; thus, we strongly recommend more detailed research to confirm and further explain such observations made herein.

## 3. Materials and Methods

### 3.1. Plant Material

**Figure 3:**
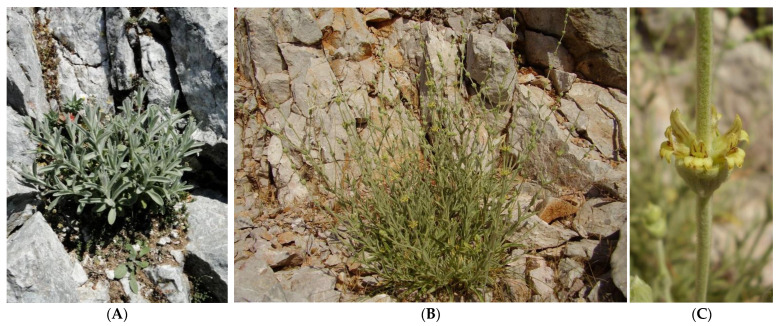
Young spring shoots (**A**) and wild-growing individual of *Sideritis sipylea* in summer bloom (**B**) showing typical yellow brown-striped bilabiate flowers arranged in remote clusters (**C**). Photographs B and C: Anastasia Stefanaki (reproduced with permission).

As *S. sipylea* (Figure 3) is considered an Endangered plant in Greece [36], parts of the above-ground shoots of 10–15 individuals of *S. sipylea* were hand-collected with caution from wild-growing populations on Mt Kerkis, Samos Island (Sample S1, at 1300 m above sea level) and on Mt Olympus, Lesvos Island (Sample S2, at 950 m above sea level), representing the most northern (Lesvos Island) and the most southern (Samos Island) distribution limit of *S. sipylea* at the eastern part of the Aegean Archipelago, Greece. Sample S1 was collected at the peak of the flowering period (mid-July 2014), while sample S2 was collected during early summer, at the beginning of the flowering period (mid-June). In both cases, the plant material used for the analysis was almost equally harvested from different individuals, thus making the collection pressure exerted during sampling almost equivalent to the routine grazing effect by herbivores and leaving untouched all basal leaves and meristems in harvested plant individuals to allow new seasonal vegetative growth. Both samples (S1, S2) were taxonomically identified, and voucher specimens were deposited at the TAU Herbarium (Laboratory of Systematic Botany and Phytogeography, Department of Botany School of Biology, Aristotle University of Thessaloniki, Greece) with duplicates at the Herbarium of the Balkan Botanic Garden of Kroussia (BBGK in Index Herbariorum), Institute of Plant Breeding and Genetic Resources, Hellenic Agricultural Organization Demeter.

### 3.2. Chemicals and Reagents

Gallic acid and 2,2-difenyl-1-picrylhydrazyl (DPPH) were purchased from Sigma Chemical Company (St. Louis, MO, USA, SAD), while *cis*-diammine dichloroplatinum (*cis*-DDP) was purchased form Tedia company (USA). All standards for GC-MS analysis were purchased form Sigma-Aldrich (Buchs SG, Switzerland). All other chemicals and reagents were of analytical reagent grade. 

### 3.3. Isolation of Essential Oil

The collected plant material was air-dried at room temperature in the dark and shade for 10 days. Then, it was grossly pulverized and each sample was subjected three times to hydrodistillation for 2 h using a modified Clevenger-type apparatus. The oil yield was expressed as ml/100 g of dry weight of the plant material (% (*v*/*w*)).

### 3.4. GC-MS Analysis

The GC/MS analyses of the EOs were performed with a Shimadzu GC-2010-GCMS-QP2010 system equipped with a split/splitless injector and a fused-silica HP-5 MS capillary column (30 m × 0.25 mm i.d., film thickness 0.25 mm). The oven temperature was programmed to rise from 50 to 290 °C at 4 °C/min (injector temp.: 230 °C; carrier gas: He (1.0 mL/min); ionization voltage: 70 eV; injection volume: 1.0 mL). The arithmetic indices (AIs) for all compounds were determined using a homologous series of n-alkanes (C9–C25) as standards [55]. The contents (relative percentages) of the separated compounds were calculated from the total ion chromatograms by a computerized integrator. The identification of the components was based on the comparison of their mass spectra with those listed in the NIST21 and NIST107 mass-spectral libraries [56] and of their AIs with literature data [57]. Whenever possible, the essential oils were subjected to chromatography with authentic compounds (Sigma-Aldrich, Buchs SG, Switzerland).

### 3.5. Biological Activities of the Studied Essential Oils

#### 3.5.1. Antioxidant Activity

Antioxidant activity was assessed using both the DPPH radical scavenging activity assay [38] with slight modifications [58] and the ABTS test [59]. Gallic acid (GA), ascorbic acid (AA) and butylatedhydroxytoluene (BHT) were used as reference antioxidants in both assays. All samples were analyzed in triplicate and the results were expressed as IC_50_ values in μg/mL.

#### 3.5.2. Cytotoxic Activity 

A standard cytotoxic activity test was performed [60] using the MTT (3-[4,5-dimethylthiazol-2-yl]-2,5-diphenyl tetrazolium bromide) assay [61,62]. The following cell lines were used (cell lines were donated from the collection of cell lines of the Institute of Virology, Vaccines and Serums "Torlak", Belgrade, Serbia): RD (cell line derived from human rhabdomyosarcoma), Hep2c (cell line derived from human cervix carcinoma–HeLa derivative) and L2OB (cell line derived from murine fibroblast), against which the activity of the obtained extracts was measured. The results were expressed as IC_50_ values (µg/mL), a threshold which was defined as the concentration of an agent inhibiting cell survival by 50% in comparison to a vehicle-treated control [63].

#### 3.5.3. Antimicrobial Activity

Antimicrobial activity was estimated by measuring the essential oils’ minimum inhibitory concentrations (MICs) against six microbial (*Staphylococcus aureus* ATCC 25923, *Klebsiella pneumoniae* ATCC 13883, *Escherichia coli* ATCC 25922, *Proteus hauseri* ATCC 13315, *P. mirabilis* ATCC 14153, *Bacillus subtilis* ATCC 6633) and two fungal strains (*Candida albicans* ATCC 10231, *Aspergillus niger* ATCC 16404). MICs of the EOs against the tested bacteria were determined by the microdilution method in 96 multi-well microtiter plates [64]. The average of three values was calculated and the obtained value was taken as the MIC for the tested sample in comparison to standard drugs (Amracin for bacteria and Nystatin for fungi) [65]. The results were expressed in μg/mL.

### 3.6. Spatial Data

All the currently accessible occurrence data of *S. sipylea* in Greece were derived from the Swedish Virtual Herbarium [66], JACQ consortium [67], GBIF Secretariat [68] and lastly from the database of the Institute of Plant Breeding and Genetic Resources of the Hellenic Agricultural Organization Demeter. The available presence data in Greece were organized in Table 6 and all duplicate data were excluded from the list. 

### 3.7. Climate Data

The climatic data used in this study were derived from Worldclim.org [69] and consisted of raster files of 1 km² analysis including arithmetic values for the monthly temperature, monthly precipitation and the 19 bioclimatic variables. The rasters underwent all necessary measure system conversions that needed to be applied. 

To extract arithmetic data for the known *S. sipylea* localities in Greece, we used the R’s raster package [70] to stack the raster collection and correspond the available coordinates to raster files’ cells. The extracted data were organized and summarized in a factsheet that illustrated the basic ecological preferences of the wild-growing populations of *S. sipylea* in their wild habitats in Greece [34].

### 3.8. Samos and Lesvos Habitat Comparison

To determine potential differences in the climate conditions prevailing in the habitats of *S. sipylea* in the Lesvos and Samos islands where the two analyzed samples originated from, we performed an independent samples *t*-test between the climatic data available for Lesvos Island and those from Samos Island. The two independent groups of the statistical tests were the Lesvos and Samos islands, while the dependent variables were the monthly temperature, monthly precipitation and the 19 bioclimatic variables. The null hypothesis was that climate factor values for *S. sipylea* wild habitats in Samos Island do not differ from the respective climate factor values in its wild habitats of Lesvos Island (see also [70]). In addition, to further validate the results of this statistical analysis, we compiled all the climate data that were extracted and used for the ecological profile in Supplementary Material Table S2 showing the mean values for Samos Island as well as the values for the single locality for Lesvos Island.

## 4. Conclusions

The results of the present study documented the diversity in the chemical composition of the essential oils and showcased the ecological preferences of the Endangered mountain tea plant species of the East Aegean area, namely *Sideritis sipylea*. We found that its essential oils mainly reflect the effect of the collection date and season (S1 and S2 plant samples collected in different stages during flowering) and the effect of geographical–environmental conditions (wild-growing plants of S1 and S2 originated from different altitudes on the two Greek islands). The diversity in the chemical profiles of these samples further influenced the concomitant variation in their biological activity. 

Although the data assessed herein may seem insufficient, we must note that the spatial data used in this study include all the currently available distribution and climatic information for *S. sipylea* in Greece. It is highly suggested for this species to be further analyzed and studied regarding its chemical composition, spatial distribution and climate preferences across its geographical range both in Greece and Anatolia of Turkey. Our study offers new ecological and pharmacognostic insight for *S. sipylea* and consolidates our knowledge for a poorly studied species in the genus *Sideritis* by revealing species-specific climate data in an ecological profile for the first time; by attempting to associate the essential oil composition with local climate data; by documenting 12 new constituents of its essential oil; by presenting for the first time the antioxidant capacity and the cytotoxic effect of its essential oils in cell lines; and by reporting on the effect of its essential oil on several microorganisms among which four had never been tested before. In this way, this study offers support for a wider utilization of *S. sipylea* in the folk medicine of the Eastern Mediterranean region and paves the road for more targeted studies regarding its chemical composition in volatiles and other secondary metabolites, new biological activity tests of different compounds and ecological profiling that may allow for future conservation translocations of selected plant materials raised ex situ. Finally, this study offers insight that can be exploited for the sustainable exploitation of *S. sipylea* as a new medicinal and aromatic crop for industrial purposes. The latter is already underway at the premises of the Institute of Plant Breeding and Genetic Resources, Hellenic Agricultural Organization Demeter where three selected clones, namely GR-1-BBGK-08,4796 from Chios Island, GR-1-BBGK-14,5792 from Samos Island and GR-1-BBGK-21,268 from Ikaria Island, have been collected directly from wild-growing populations using a special permit issued by the Greek Ministry of Environment and Energy, have been asexually propagated to date and are well-acclimatized in man-made settings. Our own future studies with these valuable materials are aimed to focus on the investigation of the chemical composition of the ex situ cultivated material of *S. sipylea* and respective comparisons with wild-growing material.

## Figures and Tables

**Table 1:** Chemical profile of *Sideritis sipylea* essential oil from wild-growing populations of Mt Kerkis, Samos Island (S1: collected at the peak of flowering) and Mt Olympus in Lesvos Island (S2: collected at the beginning of flowering), Greece (MH: monoterpene hydrocarbons, MO: oxygenated monoterpenes, SH: sesquiterpene hydrocarbons, SO: oxygenated sesquiterpenes).

Compound	AI	Content (%)		Identification with
		S1	S2	
*α*-Thujene * (MH)	924	0.7 ± 0.02	0.3 ± 0.01	AI, MS
*α*-Pinene (MH)	931	4.3 ± 0.05	37.7 ± 0.93	AI, MS, Co-GC
Sabinene (MH)	972	0.5 ± 0.01	12.1 ± 0.46	AI, MS
*β*-Pinene (MH)	976	6.3 ± 0.12	15.1 ± 0.50	AI, MS, Co-GC
*β*-Myrcene (MH)	989	20.4 ± 0.73	0.6 ± 0.01	AI, MS, Co-GC
*α*-Phellandrene (MH)	1006	2.5 ± 0.03	n.d. *	AI, MS
*α*-Terpinene (MH)	1016	6.1 ± 0.22	0.3 ± 0.00	AI, MS
*p*-Cymene (MH)	1025	2.2 ± 0.04	n.d.	AI, MS, Co-GC
Limonene (MH)	1031	3.0 ± 0.06	1.3 ± 0.06	AI, MS, Co-GC
*β*-Phellandrene (MH)	1031	3.5 ± 0.10	n.d.	AI, MS
*γ*-Terpinene (MH)	1059	2.7 ± 0.03	0.8 ± 0.02	AI, MS
Terpinolene (MH)	1085	0.3 ± 0.00	0.1 ± 0.00	AI, MS
Linalool (MO)	1101	0.7 ± 0.01	n.d.	AI, MS, Co-GC
*α*-Campholenal * (MO)	1130	0.1 ± 0.00	0.2 ± 0.00	AI, MS
*trans*-Verbenol * (MO)	1149	n.d.	0.1 ± 0.00	AI, MS
Santolinyl acetate *	1175	0.1 ± 0.00	n.d.	AI, MS
Terpinen-4-ol (MO)	1183	1.3 ± 0.04	1.5 ± 0.04	AI, MS, Co-GC
*α*-Terpineol (MO)	1198	0.8 ± 0.01	0.4 ± 0.00	AI, MS
*cis*-3-Hexenyl isovalerate	1232	0.1 ± 0.00	0.1 ± 0.00	AI, MS
Pulegone (MO)	1242	0.5 ± 0.01	0.8 ± 0.02	AI, MS, Co-GC
Carvone (MO)	1250	0.1 ± 0.00	n.d.	AI, MS, Co-GC
Bornyl acetate	1286	0.1 ± 0.00	n.d.	AI, MS, Co-GC
Thymol (MO)	1293	3.1 ± 0.08	n.d.	AI, MS, Co-GC
Carvacrol (MO)	1300	6.2 ± 0.13	0.1 ± 0.00	AI, MS
*α*-Ylangene * (SH)	1375	0.3 ± 0.01	n.d.	AI, MS
*β*-Bourbonene (SH)	1384	0.2 ± 0.00	n.d.	AI, MS
*β*-Caryophyllene (SH)	1420	11.8 ± 0.27	5.6 ± 0.51	AI, MS, Co-GC
*cis*-*β*-Farnesene * (SH)	1441	n.d.	0.3 ± 0.01	AI, MS
*trans*-*β*-Farnesene (SH)	1452	0.3 ± 0.01	0.9 ± 0.02	AI, MS
*α*-Caryophyllene (SH)	1457	0.2 ± 0.00	n.d.	AI, MS, Co-GC
*allo*-Aromadendrene (SH)	1461	0.1 ± 0.00	n.d.	AI, MS
Cumacrene * (SH)	1470	1.9 ± 0.05	0.1 ± 0.00	AI, MS
Germacrene D (SH)	1482	2.7 ± 0.07	4.2 ± 0.10	AI, MS
Viridiflorene * (SH)	1492	0.3 ± 0.01	n.d.	AI, MS
Bicyclogermacrene * (SH)	1497	7.1 ± 0.23	5.5 ± 0.16	AI, MS
*β*-Bisabolene (SH)	1508	0.1 ± 0.00	0.6 ± 0.02	AI, MS
*δ*-Cadinene (SH)	1519	0.4 ± 0.02	n.d.	AI, MS
Spathulenol * (SO)	1580	2.0 ± 0.08	1.8 ± 0.05	AI, MS
Caryophyllene oxide (SO)	1586	1.8 ± 0.06	0.4 ± 0.01	AI, MS, Co-GC
Ledol (SO)	1598	3.9 ± 0.12	n.d.	AI, MS
*α*-Cadinol * (SO)	1648	0.5 ± 0.02	n.d.	AI, MS
*α*-Bisabolol * (SO)	1689	n.d.	1.2 ± 0.09	AI, MS

* Not previously reported [23]; n.d.: not detected.

**Table 2:** Antioxidant activity in the DPPH and ABTS tests of *Sideritis sipylea* essential oils extracted from wild-growing Greek material (S1: mid-summer collection, Samos Island; S2: early summer collection, Lesvos Island) compared to reference antioxidants (GA: gallic acid, AA: ascorbic acid, BHT: butylated hydroxytoluene).

Sample	IC_50_ (µg/mL)	
	DPPH Scavenging Activity ± SD	ABTS Scavenging Activity ± SD
S1	17.48 ± 0.76	13.81 ± 3.54
S2	11.15 ± 1.13	6.28 ± 1.37
GA	3.79 ± 0.69	1.43 ± 0.93
AA	6.05 ± 0.34	9.34 ± 2.69
BHT	15.61 ± 1.26	2.12 ± 0.45

**Table 3:** Antimicrobial activity of *Sideritis sipylea* essential oil extracted from wild-growing Greek material (S1: mid-summer collection, Samos Island; S2: early summer collection, Lesvos Island) against six microbial and two fungal strains compared to reference antibiotics for bacteria (A: Amracin) and fungi (N: Nystatin).

Microbial and Fungal Strains	MIC (µg/mL)			
	S1	S2	A	N
*Staphylococcus aureus* ATCC 25923	12.50	6.25	0.97	-
*Klebsiella pneumoniae* ATCC 13883	100.00	50.00	0.49	-
*Escherichia coli* ATCC 25922	25.00	50.00	0.97	-
*Proteus hauseri* ATCC 13315	50.00	25.00	0.49	-
*Proteus mirabilis* ATCC 14153	50.00	25.00	0.49	-
*Bacillus subtilis* ATCC 6633	50.00	50.00	0.24	-
*Candida albicans* ATCC 10231	3.12	6.25	-	1.95
*Aspergillus niger* ATCC 16404	12.50	12.50	-	0.97

**Table 4:** Cytotoxic activity of *Sideritis sipylea* essential oil extracted from wild-growing Greek material (S1: mid-summer collection, Samos Island; S2: early summer collection, Lesvos Island) in one murine (L2OB) and two human cell lines (Hep2c, RD) compared to the standard cytotoxic agent *cis*-diammine dichloroplatinum (*cis-*DDP).

Sample	IC_50_ (µg/mL)		
	Hep2c ± SD	RD ± SD	L2OB ± SD
S1	23.64 ± 2.23	34.87 ± 1.20	17.51 ± 1.99
S2	43.92 ± 1.09	13.56 ± 0.67	19.49 ± 3.73
*cis*-DDP	0.94 ± 0.55	1.4 ± 0.97	0.72 ± 0.64

**Table 5:** Temperature and precipitation limits of *Sideritis sipylea* as natural adaptations shaping its occurrence in wild Greek habitats. Means were calculated based on thirteen known localities to date in Greece (N = 13).

	Mean Highest	Highest	Mean Lowest	Lowest
Temperature	27.920 ± 1.11 °C	30.7 °C	3.48 ± 1.53 °C	1.3 °C
Precipitation	136 ± 11.43 mm	154 mm	5.00 ± 1.87 mm	2 mm

**Table 6:** Compilation of all known *Sideritis sipylea* localities in Greece representing its natural distribution in four islands of the Eastern Aegean Archipelago in WGS84 coordinate system.

Localities	East Aegean Island	Longtitude	Latitude
1	Ikaria	26.13333333	37.58333
2	Ikaria	26.1500000	37.56667
3	Chios	26.03333333	38.50000
4	Ikaria	26.18333333	37.58333
5	Samos	26.600000	37.71667
6	Ikaria	26.29166667	37.61667
7	Samos	26.6500000	37.73333
8	Samos	26.6000000	37.73333
9	Chios	26.07138889	38.38417
10	Chios	26.0250000	38.52333
11	Samos	26.79583333	37.78778
12	Chios	25.98333333	38.550000
13	Lesvos	26.3500000	39.06667

## Data Availability

The data presented in this study are available on request from the corresponding authors.

## Supplementary Materials

The following supporting information can be downloaded at: https://www.mdpi.com/article/10.3390/plants12040836/s1, Table S1: SPSS-derived results and statistically significant factors (*p* < 0.05) of the independent samples *t*-test performed regarding the habitat comparison of *Sideritis sipylea* samples from Lesvos and Samos islands (Greece); Table S2: R-derived environmental values for each one of the factors studied in Samos (mean for three available locations) and Lesvos islands (a single location) confirming the *t*-test analysis.
